# Incidence variation of prostate and cervical cancer according to socioeconomic level in the Girona Health Region

**DOI:** 10.1186/1471-2458-14-1079

**Published:** 2014-10-17

**Authors:** Gemma Renart Vicens, Marc Saez Zafra, Judit Moreno-Crespi, Bernat C Serdà Ferrer, Rafael Marcos-Gragera

**Affiliations:** Research Group on Statistics, Applied Economics and Health (GRECS), CIBER of Epidemiology and Public Health (CIBERESP), University of Girona, Campus de Montilivi, Girona, 17071 Spain; Epidemiology Unit and Girona Cancer Registry, Oncology Coordination Plan, Department of Health, Autonomous Government of Catalonia, Catalan Institute of Oncology, Girona Biomedical Research Institute, Girona, Spain; Departament d’Infermeria. Universitat de Girona (UdG), Girona Biomedical Research Institute, Girona, Spain

**Keywords:** Incidence, Prostate cancer, Cervical cancer, Besag, York and Mollie model, Deprivation index, Mutual standardization problem

## Abstract

**Background:**

The main aim of this study, using a spatial-temporal model, is to analyse the link between a deprivation index and the incidence of prostate and cervical cancer in the Girona Health Region (GHR).

**Methods:**

This is a population-based study which includes all the inhabitants in the GHR in the period 1993–2006. In order to assess prostate/cervical cancer risk, Besag, York and Mollie (BYM)’s spatial-temporal version of the model was used and four random effects were introduced: (non-spatial) unstructured variability, spatial dependency, temporal dependency and spatial-temporal interaction. As an explanatory variable, a deprivation index was introduced at the census tract level. Furthermore, the percentage of the population between 45–64 years of age and over-65 was also considered as explanatory variables.

**Results:**

In the case of prostate cancer, all the variables which were introduced into the model showed a significant correlation with the relative risk, except for the second quintile of the deprivation index. Furthermore, as the index increased the correlation became negative and lower. Thus, the correlation between the relative risk and the two age bands proved to be lower, the higher the age was. In the case of cervical cancer, only the correlation between the over-65 age band and the relative risk was found to be statistically significant and positive.

**Conclusions:**

In the case of prostate cancer, the results obtained in the GHR are in line with similar analyses. However, in the case of cervical cancer, no significant relationship between incidences in this location or economic status was found.

## Background

The study of incidence and mortality in small areas and their relationship with different socioeconomic indicators has recently been attracting a growing interest in different countries [1–6]. Not only can individual factors explain the causes of the disease, but so too must the contextual factors of the area of residence be taken into account, particularly in small areas, as those characteristics may contribute to the socioeconomic and environmental differences in health.

In the case of cancer, there are various studies which show how the contextual factors of place (i.e. area of residence) can have an effect not only the incidence of cancer, but also on mortality rates. It would appear that the most disadvantaged areas tend to have higher mortality rates [1, 3, 5–8].

This study focuses on this relationship for the particular cases of prostate cancer and cervical cancer. According to Ferlay et al., 2013 [9] when both sexes are combined prostate cancer is the fourth most common cancer and the second most frequently occurring cancer in men, while cervical cancer is the fourth most commonly occurring cancer in women and the seventh most frequent overall.

Prostate cancer is the second most common cancer in European men and represents approximately 12.1% of all newly diagnosed cancer cases [9]. In Europe, incidence rates vary greatly. The highest rates were estimated in northern and western European countries (such as Norway and France) and the lowest in the central and eastern European countries (e.g. Republic of Moldova and Albania) [9].

In Spain prostate cancer, ahead of lung cancer, is the most common tumour found in men. Approximately 20,000 cases per year are diagnosed, which represents 21% of all tumours among men [9]. The estimated incidence rate for the Spanish population in 2012 is 96.8 cases per 100.000 males [9] and so is similar to other developed countries. In the case of the GHR, the incidence rate is estimated to be 97.6 cases per 100 000 males per year [10, 11].

In the majority of cases, a diagnosis of prostate cancer occurs between 60 and 80 years of age, although there are a significant number of cases from 50 years of age onwards, with the average age being 69. In general, prostate cancer may be considered to be a tumour which is more typical in older men [12].

Since the introduction of the Prostate Specific Antigen screening in Spain, the rate of diagnosis has increased significantly but the specific mortality rate has in fact decreased [13–17].

Cervical cancer is the fourth most common neoplasia among women in the world today and represents a seventh of all neoplasias [9]. As in the case of prostate cancer, there are significant differences between more and less developed countries: while in the latter case it is, after breast cancer, the second most commonly occurring tumour, in developed countries its frequency has decreased significantly in recent decades thanks to prevention and early detection strategies and campaigns [18]. 83% of the cases of cervical cancer diagnosed each year occur in developing countries [19], and are, ahead of breast cancer, the most frequent cause of death from cancer in those countries. The estimated incidence for the Spanish population is 7.69 per 100 000 women [18].

The average age at diagnosis is 48 years old, although approximately 47% of women with cervical cancer are diagnosed before the age of 35, and only 10% are diagnosed in women over the age of 65 [10].

In Spain, approximately 2 100 cases are diagnosed annually; representing 3.3% of cancers in women and with 7.6 new cases/100 000 women/year the incidence of cervical cancer may be considered one of the lowest in the world [20].

The main aim of this study, using the spatial-temporal version of the Besag, York and Mollie model [21, 22], is to analyse the variation in incidence of both neoplasias according to the area of residence and in relationship to socioeconomic deprivation in the Girona Health Region (GHR).

## Methods

### Data setting

The present study was undertaken within the framework of the MEDEA project and one of the objectives was to estimate the relative risks associated with a deprivation index for various cancer locations in the Girona Health Region (GHR), (which basically coincides with the Province of Girona, in the north of Catalonia, Spain), and to ascertain whether this deprivation index could explain part of the spatial variability found in some of these locations [6, 23].

All the residents in the GHR (which according to the 2006 municipal population register were 670 096 inhabitants of whom 339 839 were males and 330 257 females) were considered as a study population. The study took place from 1993 to 2006, both inclusive, and the geographical area of analysis was the census tract.

In this paper, the analysis was performed on data provided by the Girona Cancer Registry [24, 25] for incident cases of prostate cancer (ICD-10: C61) in men, and cervical cancer (ICD-10: C53) in women.

To capture the specific socioeconomic contextual effects of geographic location on health, a deprivation index (DI) at the census tract level was introduced into the model as an explanatory variable. The DI was constructed by aggregating sixteen socio-economic variables, available in the Spanish Census of Population and Housing, 2001, using the DP_2_ method, an iterative procedure that weights partial indicators depending on their correlation with the global index. Details may be found in Salcedo et al*.*[26].

### Statistical analysis

In order to explain the variation of incidence in cases of both prostate and cervical cancer, a full Bayesian perspective was followed and a spatial-temporal version of the Besag, York and Mollie model [21, 22] (BYM) was used. In particular, a probability distribution (prior distribution) was assigned to the parameters and in order to gather together all the unexplained variability, four random effects were introduced into the model: i) (non-spatial) unstructured variability, ii) spatial dependency, iii) temporal dependency and iv) spatial-temporal interaction. It should be noted that separability between spatial and temporal patterns was assumed and interaction between the two components was allowed.
1

where the subscript *i* denoted the census tract (i = 1,…,542); *t* the year (1993,…,2006); μ_it_ was the mean of the observed cases, E(O_it_); O_it_ was the number of cases observed in each census tract *i* and year *t*; Pob_it_ denoted the population of the census tract *i* and year *t*; Q.index_j_ denoted a dummy variable relative to the quintile *j* of the deprivation index, for each census tract *i* and year *t* (the first quintile was taken as a reference category). P4564_it_ was the percentage of the population between 45 and 64 (both inclusive); and P65M_it_ the percentage of the population over 65 (both in the census tract *i* and year *t*). The group aged under 44 did not appear in the model in order to avoid problems of co-linearity with the rest. Finally, *βs* and *γs* denoted unknown parameters. Here, the parameters of interest were *β*_*j*_, or better, *exp(β*_*j*_*)*, the relative risks associated to the quintiles of the deprivation index.

It should be noted that in the specified model the number of cases expected in the census tract were not used as an offset, but rather the population (men or women) of the same. This is because unlike the standard BYM model, here, the crude incidence rate (from the census tract) was used as an indicator of mortality and not the standardised incidence ratio. The reason for this was to avoid the so-called ‘mutual standardisation’ problem [26, 27]. Rosenbaun and Rubin [28] show how the use of standardised rates as the response variable in ecological regression models leads to biased results if only the answer, and not the predictor, is adjusted for the same confounder, usually age distribution. When the predictor is not adjusted, it is implicitly assumed that its effect is constant for all strata of the confounding variable. Grisotto et al. [29], in line with Rosenbaun and Rubin [28], show that unbiased estimators can be obtained by adjusting the response and predictors (the index of deprivation in this case) with the same variable (age distribution) or, even more straightforwardly, by using crude rates as the response variable and entering age (as an average or structured) as an explanatory variable of the model. This is why the age structure of the census tract (proportion of men and women aged 45 to 64 and 65 years or over) was introduced. The introduction of age also enabled its effect in the model to be controlled [26].

With random effects: υ_ι_ denoted heterogeneity and captures the spatially unstructured variation of relative risks. It was made up of zero-mean independent Gaussian random variables on *i* (census tract), with a constant variance,. Si was the random effect which captures the spatial variability and to do this a parametric model was established. Specifically, and following the recent work of Lindgren et al. [30], a Matérn structure [31] was established for spatial dependence:


where *h* denoted the Euclidean spatial distance between the centroids of census tracts *i* and *j*;  was the variance of the spatial structured term; *K*_*ν*_ denoted the modified Bessel function of the second kind and order *ν* >0, with *ν* as the smoothing parameter. In this paper *ν* in 1 was fixed. *κ* (*κ* >0) was a scaling parameter related to the range, in other words, the distance at which the spatial correlation becomes almost nil.

Both the temporal dependence, *τ*_*t*_ (on *t*, i.e. the year) and the spatial-temporal interaction, *η*_*it*_ (on *j* and *t*) were assumed smoothed functions for Gaussian vectors of random variables, in particular random walks of order 1 and RW1 were constructed assuming independent increments [32]:


The density for x is derived from its n-1 increments as:


where Q = τR and R is the structure matrix reflecting the neighbourhood structure of the model.

Typically, in a standard BYM, a Poisson model is assumed for approximating the distribution of the count observation. In this case, however, the data generating process resulted in a larger number of zero counts than would be expected (in the case of prostate cancer there were 54.5% observed zero counts vs. the expected 35.1%; assuming the same mean). If this were the case, the dispersion of the Poisson model would underestimate the observed dispersion. Thus in the case of prostate cancer, a mixed-distribution model was also used, specifically a zero-inflated Poisson (ZIP) [33, 34]. In the case of cervical cancer, there were 94.4% zero counts observed, 5.4% of the census tracts with one count and only 0.2% of the census tracts with two counts. For this reason, it was considered that for cervical cancer we had a dichotomous response, (zero counts vs. at least one count) leading to a binomial distribution.

For Bayesian computation, that is to say for obtaining the marginal posterior distributions for each of the elements of the parameters vector, the INLA approach [35] was used. In short, a Gaussian Markov random field (GMRF) representation was constructed explicitly from a stochastic partial differential equation (SPDE) whose solution is a Gaussian field (GF) with a Matérn covariance function [30]. Rather than using a regular lattice, as is standard practice and which would imply an estimate with a high computational cost and very little efficiency [30], a Matérn spatial covariance structure in a triangulation (triangulation of Delaunay [36]) of the GHR was specified, i.e. with very low computational cost and, most importantly in this context, much greater efficiency.

All analyses were conducted using the free software environment R (version 2.14.2) [37] and INLA [32, 35] (integrated nested Laplace approximation).

A natural way to compare models is to use the criterion based on a trade-off between the fit of the data to the model and the corresponding complexity of the model. Deviance Information Criterion (DIC) is the Bayesian model comparison criterion based on this principle) [38]:


where  is the deviance evaluated at the posterior mean of the parameters and *p*_*D*_ denotes the ‘effective number of parameters’ which measures the complexity of the model [38]. DIC may under penalise complex models with many random effects [39, 40]. For this reason the conditional predictive ordinate (CPO) [41, 42] was also used. CPO expresses the posterior probability of observing the value (or set of values) of *y* when the model is fitted to all data except *y*_*i*_, *CPO*_*t*_ = *π*(*y*_*t*_^*obs*^|*y*_- *t*_) (*y*_- *t*_ denotes the observations y with the i-th component omitted). This facilitates the computation of the cross-validated log-score, cv.ls, (*cv.ls = -(mean(log(cpo)))*) [43], for model choice. Both the lower DIC and the lower cv.ls involve the best model.

## Results

The results obtained in the estimations of the models outlined above are shown in Tables 1 and 2 in the case of prostate cancer, and in Table 3 for cervical cancer. In each case, Estimation 1 corresponds to the model (1) without the spatial-temporal interaction and Estimation 2 corresponds to the model with interaction. As has been previously mentioned, in the case of prostate cancer, different estimations have been carried out depending on whether the Poisson model or the zero-inflated Poisson model was used (Tables 1 and 2, respectively).

**Table 1:** **Results of the Bayesian computation- prostate cancer**

Prostate cancer (men)		
	ESTIMATE 1*	ESTIMATE 2*
	Mean (95% credibility interval)	Mean (95% credibility interval)
RR_deprivation_		
RR_Q2-deprivation_	0.8592 (0.7008, 1.0051)	0.8625 (0.7036, 1.0055)
RR_Q3-deprivation_	**0.8129 (0.6618, 0.9980)**	**0.8136 (0.6624, 0.9991)**
RR_Q4-deprivation_	**0.7315 (0.6025, 0.8878)**	**0.7308 (0.6016, 0.8872)**
RR_Q5-deprivation_	**0.5768 (0.4771, 0.6969)**	**0.5766 (0.4769, 0.6968)**
Log RR Population		
Pop. 45–64 years old	**3.8208 (1.0350, 6.6063)**	**3-9326 (1.1603, 6.7041)**
Pop. 65 years old or older	**2.4520 (0.4642, 4.4275)**	**2.4266 (0.4522, 4.3879)**
Random effects standard error		
Temporal dependency	0.03028	0.02958
Heterogeneity	0.02954	0.03083
Spatial dependency	0.09561	0.09623
Spatial-temporal interaction	-	0.00201
DIC	10724.91	10729.93
-log (mean (cpo))	1.0689	1.0695

Shaded, the 95% credibility interval did not contain the unit (i.e. statistically significant at 95%).

*Estimation 1: Model with separability between spatial and temporal patterns and without interaction between these.

Estimation 2: Model with separability between spatial and temporal patterns and with interaction between these.

**Table 2:** **Results of the Bayesian computation- prostate cancer**

Prostate cancer (men)		
	ESTIMATE 1*	ESTIMATE 2*
	Mean (95% credibility interval)	Mean (95% credibility interval)
RR_deprivation_		
RR_Q2-deprivation_	0.8152 (0.6298, 1.0527)	0.8198 (0.6326, 1.0598)
RR_Q3-deprivation_	0.8030 (0.6190, 1.0393)	0.8063 (0.6208, 1.0446)
RR_Q4-deprivation_	**0.7441 (0.5845, 0.9473)**	**0.7454 (0.5848, 0.9499)**
RR_Q5-deprivation_	**0.6000 (0.4712, 0.7622)**	**0.5998 (0.4706, 0.7627)**
Log RR Population		
Pop. 45–64 years old	2.7372 (-1.3453, 6.8193)	2.9080 (-1.1261, 6.9473)
Pop. 65 years old or older	2.5813 (-0.6199, 5.7470)	2.4672 (-0.6904, 5.5819)
Random effects standard error		
Temporal dependency	0.04279	0.00675
Heterogeneity	0.04576	0.04519
Spatial dependency	0.03810	0.04213
Spatial-temporal interaction	-	0.00204
DIC	11289.16	11293.09
-log(mean(cpo))	1.1239	1.1244

Shaded, the 95% credibility interval did not contain the unit (i.e. statistically significant at 95%).

*Estimation 1: Model with separability between spatial and temporal patterns and without interaction between these.

Estimation 2: Model with separability between spatial and temporal patterns and with interaction between these.

**Table 3:** **Results of the Bayesian computation- cervical cancer**

Cervical cancer (women)		
	ESTIMATE 1*	ESTIMATE 2*
	Mean (95% credibility interval)	Mean (95% credibility interval)
RR_deprivation_		
RR_Q2-deprivation_	0.4114 (0.0251, 6.7160)	0.4067 (0.0247, 6.6799)
RR_Q3-deprivation_	0.9395 (0.1042, 12.9488)	0.9485 (0.1049, 13.1051)
RR_Q4-deprivation_	0.8123 (0.1136, 9.6775)	0.8143 (0.1136, 9.7240)
RR_Q5-deprivation_	1.1477 (0.1680, 13.1919)	1.1360 (0.1659, 13.0802)
Log RR Population		
Pop. 45–64 years old	22.9553 (-15.4972, 63.1097)	22.6265 (-15.8452, 62.9551)
Pop. 65 years old or older	**22.1030 (6.8930, 34.5706)**	**22.3107 (6.9830, 34.9207)**
Random effects standard error		
Temporal dependency	0.00628	0.00636
Heterogeneity	0.00637	0.00650
Spatial dependency	0.05099	0.05210
Spatial-temporal interaction	-	0.00447
DIC	2288.77	2289.19
-log(mean(cpo))	0.2223	0.2224

Shaded, the 95% credibility interval did not contain the unit (i.e. statistically significant at 95%).

*Estimation 1: Model with separability between spatial and temporal patterns and without interaction between these.

Estimation 2: Model with separability between spatial and temporal patterns and with interaction between these.

In general it can be noted how the fit in both calculations for all cases (DIC and CPO) is practically identical, although it is slightly better in Calculation 1. In addition, in the case of prostate cancer (Table 1) and in the case of cervical cancer (Table 3), it can be observed how both the deviation of the random effect, which captures the spatial variability, and the standard deviation of the random effect, which captures the unstructured variability, behave in a similar way. However, in the case of prostate cancer (Table 2) it has a different pattern. The deviation of the random effect, which captures the unstructured variability, is slightly lower for Estimation 2.

It can also be observed that for prostate cancer the significant relative risk statistic associated with the quintiles of the deprivation index and the age ranges introduced into the model do not vary between the two estimations in each model (Tables 1 and 2); however, there are differences between both models.

In the first case (Table 1), all the explanatory variables were significant except for the second quintile of the deprivation index. It should be noted that the association of the relative risk with this variable is negative and decreases as the deprivation index increases. The association of the relative risk with the age ranges included in the model is also statistically significant; namely being lower at higher ages. In the second case (Table 2), considerable differences are evident in this respect, with the only statistically significant association being the association of relative risk with the fourth and fifth quintiles of the deprivation index. However, this relationship maintains the same trend observed in the previous case: the higher the deprivation index, the lower the relative risk.

In the case of cervical cancer (Table 3), in terms of the statistical significance of the explanatory variables with the relative risk, the results obtained for both estimations are identical; that is, only the correlation between the over-65 age range and the relative risk is significant and positive.

## Discussion and conclusions

The results of this paper show that the higher the deprivation, the lower the risk of incidence of prostate cancer. However, in the case of cervical cancer the geographical variability of its incidence is not explained by deprivation.

With the statistical results, while the fit is slightly better in the models without spatial-temporal interaction, the estimated relative risks are practically the same in the models with or without interaction. In fact, in all cases, spatial dependency dominates, with a typical deviation practically twice that of temporal dependency. This may mean that the spatial incidence pattern for both types of cancer has remained stable during the period under consideration.

In reference to the incidence of prostate cancer, while very few, there are other studies which have also found that relative risks associated with deprivation are lower than unity [44–47]. This could be related with the stage and grade of the cancer at diagnosis, which is more advanced in areas with higher levels of deprivation. In fact, it has been reported that there is lower participation in opportunistic screening for prostate cancer via prostate specific antigen (PSA) by those from a lower socioeconomic level [11, 48]. However, no differences in PSA levels [48] nor in mortality for prostate cancer were found [8, 48].

In contrast, cervical cancer (statistically significant) affects more women from lower socioeconomic levels [49, 50]. In our case, while we have not been able to find a statistically significant relationship, we did find a significantly higher relative risk than unity for very high levels of deprivation. Some causes of this could be related to increased sexual activity among women in areas with high levels of deprivation and/or to the immigration of women coming from countries which have a high prevalence of the viral infection human papillomavirus.

As an ecological design was used, this can be considered the main limitation of this work and therefore the usual caveats must be taken into account. In this sense, while the methods applied in ecological regressions may describe the spatial-temporal distribution of incidence, they do not explain why the risk is higher in some small areas. For this, further studies with individual data which evaluate the risk factors of specific causes of incidence are needed [21]. In fact, it is well known that in ecological studies a relationship between exposure and the factor risk studied cannot be obtained since it is not known whether individuals more exposed to a certain factor in each geographic area are in the case study. In our case however, we did not have any individual-level socioeconomic indicator to verify this fact. This represents a significant limitation since, at least for the incidence of prostate cancer, independent effects of socioeconomic status at the individual level, and deprivation, in terms of geographical area (ecological level) have been found [44, 45, 47].

In short, we found that deprivation may in fact limit access to health services, resulting in a late diagnosis and therefore an increase in the incidence of prostate cancer. Thus, putting screening and prevention programs into place could reasonably contribute to reducing health inequalities; at the very least in the case of prostate cancer incidence.

### Ethics statement

Ethical approval from an Ethics Committee was not required for this study.
